# Claparède (1904) on Monocular Stereopsis: History, Theory, and Translation

**DOI:** 10.1177/2041669517731411

**Published:** 2017-09-20

**Authors:** Robert P. O’Shea

**Affiliations:** Discipline of Psychology, School of Psychology and Exercise Science, 5673 Murdoch University, Perth, Australia; Discipline of Psychology, School of Health and Human Sciences, Southern Cross University, Coffs Harbour, Australia

**Keywords:** three-dimensional perception, binocular vision, cue combination, depth, perception, stereopsis, history, monocular vision, Claparède

## Abstract

Astoundingly, looking at a photograph with one eye can yield an experience of depth of the depicted objects similar to that from viewing the real objects with both eyes. Édouard Claparède (1873–1940) was one of the first to report this phenomenon in a French paper published in 1904. I give some historical and theoretical context to the phenomenon, provide some biographical information about Claparède, and provide a translation into English of Claparède’s paper.

## Introduction

Sometimes when we look with one eye at a flat depiction of an object, such as a photograph, we can see the object as having a palpable solidity similar to when we look at it with both eyes (e.g., Koenderink, van Doorn, & Kappers, 1994; Vishwanath & Hibbard, 2013). In order to experience this remarkable illusion, the head must be stationary; moving it would immediately reveal the flatness of the depiction from the absence of motion parallax (e.g., Wheatstone, 1838). Koenderink et al. fixed their observers’ heads with a forehead and chin rest; Vishwanath and Hibbard fixed their observers’ heads by having them look through a small aperture; this also likely promoted the illusion by obscuring the depiction’s edges (e.g., Eby & Braunstein, 1995).

The experience of palpable solidity from one eye was called “paradoxical monocular stereoscopy” by Claparède (1904, p. 465) who wrote one of the earliest papers on the topic. My aims are to set Claparède’s (1904) French-language paper into its historical and theoretical context, to give some biographical details of Claparède, and to provide a translation of the paper.

I also need to clarify some terminology. Although Claparède referred to *stereoscopy*, we would now call this *stereopsis*. Wheatstone (1838) referred to his device for seeing the illusion of depth from two flat pictures showing binocular parallax as a “stereoscope” (p. 374), so it is perhaps inevitable that using a stereoscope, and the experience of that kind of depth perception, would first be called stereoscopy (e.g., Lonie, 1856, p. 13). The earliest mentions I have been able to find of “stereopsis” in the modern sense did not occur until more than a decade after Claparède published his paper (e.g., Howard, 1919; Wilmer, 1919). (See O’Shea, 2017, for the details.)

Vishwanath (2014) has argued that the modern sense of stereopsis should be reserved for describing the experience of palpable solidity, noting that stereopsis has become synonymous with binocular stereopsis. Koenderink (1998) pointed out that the linguistic and theoretical hegemony of stereopsis began with Wheatstone’s (1838) notion that binocular stereopsis is the only source of information that yields sure, quantitative depth. I think Wheatstone was reflecting Leonardo da Vinci (1452–1519) who maintained that something special arose from using two eyes to view objects that could never be produced with one (Wade, Ono, & Lillakas, 2001).

I will go along with Vishwanath (2014) in reserving *stereopsis* for “the characteristically vivid impression of tangible solid form, immersive negative space and realness … that obtains under certain viewing and stimulus conditions” (p. 153). I will qualify the noun with the adjectives *monocular* and *binocular* to refer to viewing with one or two eyes, respectively. I will refer to the impression of solidity of an object arising from two appropriate flat depictions of it when viewed via a stereoscope as *stereoscopic vision* (cf. O’Shea, 1983)*.*

## Historical and Theoretical Context

Various people have commented on the realistic appearance of depictions of objects viewed with one eye or have developed theories about it. I list them chronologically.

### Brunelleschi (c. 1415)

We can probably mark the modern start of realistically depicting three-dimensional objects and scenes on flat surfaces with Filippo Brunelleschi (1377–1446) in about 1415. According to Blumberg (2016), Brunelleschi devised a geometrical method involving linear perspective, based on Euclidean optics (Burton, 1945) that was widely adopted. It was set down by Alberti (1435/1970) who dedicated an edition of his book to Brunelleschi. Manetti (c. 1489/1970) recorded that Brunelleschi demonstrated his method by producing a painting (now lost) of the Baptistery of Saint John in Florence as viewed from the door of the cathedral.

An observer at the cathedral door looked from behind Brunelleschi’s painting through a small hole (about the size of a lentil; Manetti (c. 1489/1970, p. 44) in its center at a reflection of the painting in a mirror he or she held or at the real baptistery, allowing the observer to compare the two. An observer “felt he saw the actual scene when he looked at the painting” (p. 44). If there were any theory underlying Brunelleschi’s observations, it was that linear perspective can yield a veridical experience of the reality of a depicted object. Brunelleschi’s small aperture compelled viewers to use one eye when looking at the real baptistery, preventing binocular stereopsis. It also created the conditions for monocular stereopsis when looking at the painting.

### Leonardo da Vinci (c. 1508)

In about 1508, Leonardo da Vinci stated, “the picture seen with one eye will place itself in relief like the actual relief, having the same qualities of light and shade” (Quaderni III 8 r; MacCurdy, 1955, p. 856). Yet, as I have already said, da Vinci also said: “It is impossible for a painting, even though executed with the greatest perfection of outline, shadow, light, and color, to seem in the same relief as the natural model, unless that natural model is looked at from a great distance with one eye” (quoted by Wade et al., 2001, p. 231).

It is likely that da Vinci’s influence on scholars who thought about the origin of the experience of depth was to accord a special status to binocular vision. This was abetted by Molyneux’s (1692) various demonstrations that we use both eyes together:

And as a Conclusion to the whole shall only add one Experiment that Demonstrates we see with both Eyes at once; and ’tis, that which is commonly known and practised in all Tennis-Courts, that the best Player in the World Hoodwinking one Eye shall be beaten by the greatest Bungler that ever handled a Racket. (pp. 293–294)

### Wheatstone (1838)

Wheatstone (1838) discovered binocular stereopsis, inventing the stereoscope to prove that binocular disparity, the differences in the images received by each eye from their different positions on the head, can yield the illusion of depth from two flat pictures shown one to each eye. According to Koenderink (1998), Wheatstone convinced many scholars to believe that binocular stereopsis is the primary, if not the only, process from which veridical depth is experienced.

Wheatstone did report monocular stereopsis; he said:

Every one must be aware how greatly the perspective effect of a picture is enhanced by looking at it with only one eye, especially when a tube is employed to exclude the vision of adjacent objects, whose presence might disturb the illusion. Seen under such circumstances from the proper point of sight, the picture projects the same lines, shades and colours on the retina, as the more distant scene which it represents would do were it substituted for it. (pp. 380–381)

He added, in keeping with his stance that only binocular stereopsis affords real depth, “The appearance which would make us certain that it is a picture is excluded from the sight, and the imagination has room to be active” (p. 381).

Of course, the other important historical development around this time was the invention of photography (Newhall, Grundberg, Rosenblum, & Gernsheim, 2016). This removed from artists the necessity to use Brunelleschi’s geometrical approach, or various tricks such as Alberti’s window (e.g., Edgerton, 2006), to make accurate two-dimensional representations of objects and scenes. Photography essentially replaced Alberti’s window with a photosensitive surface. Wheatstone himself was an enthusiastic user of photography (Wade, 2014).

Wheatstone’s explanation of why we see a flat depiction as flat with two eyes and as showing depth with one eye is consistent with a theory that has come to be known as *cue-coherence theory* (e.g., Blundell, 2015; Vishwanath & Hibbard, 2013): That depth can be experienced providing the various sources of information (cues) specifying depth are in agreement, otherwise the strongest source wins out. For Wheatstone that strongest source was binocular disparity.

### Emerson (1863)

Emerson (1863) stated that “paintings ought to be observed by one eye and from one point of view to obtain the maximum effect [of depth]” (p. 11). Yet he too, like Wheatstone (1838), distinguished between real depth afforded by binocular stereopsis and something else afforded to one eye by perspective.

### Claparède (1904)

As we shall see, in a paper comprising five paragraphs, Claparède (1904) set forth the perceptual reality of monocular stereopsis, explained that it arises from association with experienced distance of objects, explained that it disappears when the depiction is viewed with both eyes from absence of binocular parallax and of changes in convergence. Claparède is also remarkable for implying that binocular disparity is not necessarily preeminent among depth cues.

### Others up to 1941

Eaton (1919) reported that when he looked at ordinary photographs with one eye through a biconvex lens, he experienced monocular stereopsis that was similar to that from binocular stereopsis. Eaton also stated that binocular disparity is not preeminent among depth cues, anticipating later research showing that binocular disparity can be modified or even overcome by other depth cues (e.g., Blackburn & O’Shea, 1993; Johnston, Cumming, & Parker, 1993).

Wells (1920), in a review of stereoscopic vision, noted that perceived solidity of objects could also arise from viewing appropriate pictures of them with one eye. To describe this, he reflected Claparède’s terminology in referring to “monocular stereoscopic vision” (p. 12245).

According to Ames (1925), Streiff (1923) made his own detailed phenomenological observations of monocular stereopsis, citing Claparède (1904). This motivated Ames (1925) to make his own review and observations, reiterated by Schlosberg (1941). Both of them confirmed monocular stereopsis; Schlosberg said the “depth that can be obtained monocularly is very striking, and must be seen to be appreciated” (p. 601). Both reported that viewing an image through a small aperture, with altered accommodation (such as through a magnifying glass), or by reflection from a mirror, promoted the depth. Their explanations were along the lines of cue-coherence theory.

### Modern Authors

Adams (1972) showed participants a perspective image of a black-and-white-tiled floor, surrounded by a flat frame, and asked them to adjust the size and shape of quadrilaterals on a vertical wall at the rear of the image to equal those on the floor. Different participants viewed the displays from one of three different viewing distances in one of three viewing conditions: binocularly, monocularly through a 6-mm hole, or monocularly through a 0.75-mm hole. Adams found that although viewing distance yielded effects that were less than expected from a strict application of the geometry of perspective, and went in the direction of size constancy assuming the floor was horizontal, viewing condition affected only the variance of settings, with binocular viewing yielding higher variance then the two monocular conditions. He attributed this to the competing influence of flatness that binocular vision afforded, which affected different participants differently.

Koenderink et al. (1994) showed their participants monochrome photographs of objects and had them adjust a superimposed, red, measuring stimulus comprising a disk and a central line orthogonal to circle (rather like a spinning top) such that the disk appeared pictorially as if it were painted onto the surface on the object and the axis appeared normal to that surface. Participants viewed the display binocularly, monocularly, and synoptically (i.e., identical images to the two eyes with parallel lines of sight). Koenderink et al. found that binocular viewing yielded the least depth and monocular and synoptic viewing yielded more. They also concluded in favor of cue-coherence theory.

Vishwanath and Hibbard (2013) conducted three studies comparing monocular and binocular viewing of various pictures, including full-color photographs of natural objects and black-and-white synthetic images of randomly dotted, opaque cylinders. In their first study, they asked participants to report verbally or in writing about their phenomenological impressions of photographs viewed binocularly so that the frame of the photographs and the surrounding apparatus and room was visible and viewed monocularly through an aperture that restricted visibility to about 85% of the a central, approximately horizontally pitched oval area of the photographs. They conducted a content analysis to show that monocular-aperture viewing yielded stereopsis, whereas binocular viewing yielded flatness.

One theory of depth perception is that its quantitative value is determined by some Bayesian combination of depth information from all sources (e.g., Bülthoff & Mallot, 1988; Landy, Maloney, & Young, 1995; Nakayama & Shimojo, 1992): *cue-coherence theory.* Vishwanath (2014), however, produced a new theory to account for phenomena such as monocular stereopsis. It is that various depth cues sum in some way to give relative depth information. Stereopsis arises when relative depth is scaled to absolute depth via a high-precision estimate of the distance of an object from an observer.

## Who Was Claparède?

Information in this biography has been gleaned from *Encyclopædia Britannica* (2016), from Stiker (2006), Pillsbury (1941), and Claparède’s autobiography (Claparède, 1930).

### Overview

**Figure 1.**
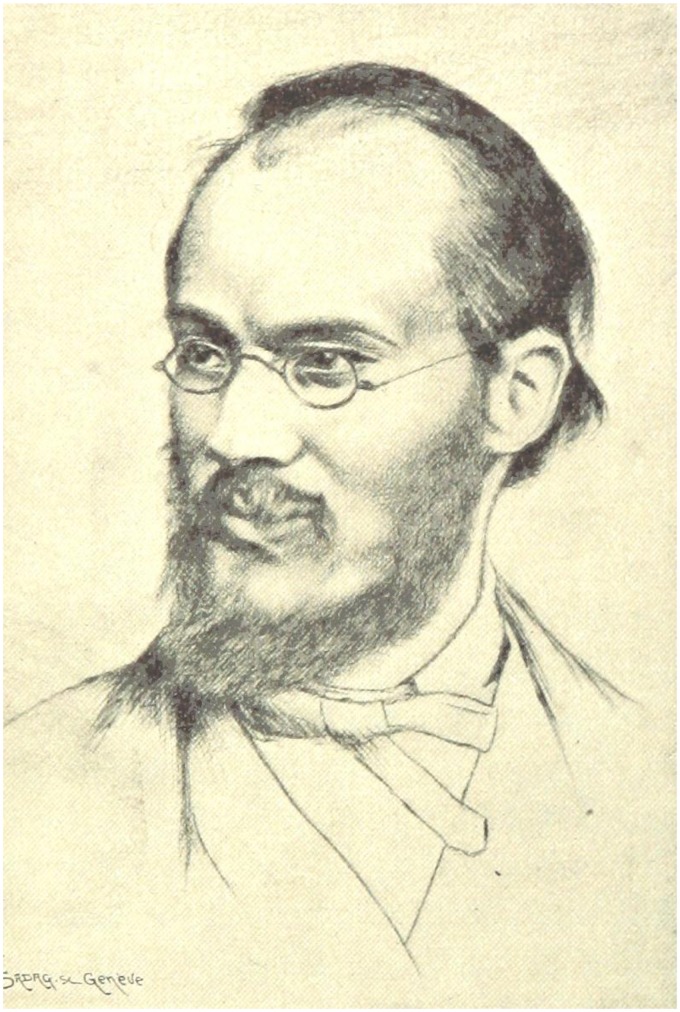
Sketch of Claparède (Studer, 1900, p. 265).

Édouard Claparède (Figure 1) was an eminent Swiss psychologist, whose notable contributions included founding and editing *Archives de Psychologie* in 1901, founding a school, the Jean-Jacques Rousseau Institute, in 1912, guiding the International Congress of Psychology from 1926, and influencing pedagogy. He also made important contributions to research into concept formation, problem-solving, animal behavior, and sleep. Claparède published more than 600 papers in psychology and at least six books.

### Life

Claparède was born in a house in the Champel neighbourhood of Geneva on March 24, 1873, the youngest of five children. He lived in that house, on and off, until his death on September 29, 1940. His ancestors had come to Geneva in 1724 after the Revocation of the Edict of Nantes legitimized persecution of his family’s Protestant religion. His father was a clergyman. In 1896, Claparède married Helene Spir, with whom he had been in love since he was 15; they had two children.

### Path to Psychology

At age 4, Claparède identified with an epynomous uncle, much talked about in his family, who had died 2 years before Claparède’s birth. That Édouard had been a prominent naturalist; Claparède determined to become a zoologist. At the age of 15, after Claparède’s father died, he determined to compromise with his father’s wish that he become a clergyman by becoming a medical missionary. A year later in 1889, Claparède finished school at the Geneva College, wrote his first published work (on how to reform the school for better education), and went to the University of Geneva to study natural sciences and medicine. There he made contact with his cousin, Théodore Flournoy, professor of psychology. Attending a public lecture by Flournoy on Wundt and Fechner’s psychology convinced Claparède he had found his vocation.

In 1891, Claparède became a member of Flournoy’s laboratory of experimental psychology. He also discovered that he was a colored-hearing synesthete; Flournoy helped him draft a questionnaire on synesthesia and to circulate it among students and staff. Claparède visited Alfred Binet in Paris who was also researching synesthesia.

In 1892, Claparède spent one semester at the University of Leipzig, working in the laboratory of Wilhelm His, Senior, who at the time was researching the anatomy of the nervous system. Claparède also briefly attended one of Wundt’s courses given by Külpe until he was excluded for applying too late. He returned to Geneva and concluded his medical studies in 1892 with an MD thesis on a patient with hemiplegia and ataxia.

### Career

After 1892, Claparède worked as a doctor, neurologist, researcher, and educational psychologist. In 1899, he was appointed as a docent at the University of Geneva and began teaching sensation and perception. In 1904, Flournoy put Claparède in charge of his laboratory. Claparède may be the first to stage intrusions into his classes to test students’ memories of these events. In 1908, Claparède was appointed as an associate professor of psychology, a position he held until his death.

### Interests in Perception and Neuroscience

Among Claparède’s numerous interests, he had an abiding interest in perception and what we now call neuroscience. Claparède worked on weight illusions, monocular stereopsis, the moon illusion, among other topics such as hypnosis and memory.

## The Translation

Physics and natural history society of Geneva

*October 6th, 1904 session*

Paradoxical monocular stereoscopy.

Mr. Ed. [Édouard] Claparède.—Everyone knows that the relief of objects and perception of depth are strongly attenuated, if not suppressed, in monocular vision. However, if instead of looking at real objects, one looks at objects that are represented on a flat surface, the opposite happens; monocular vision is stereoscopic, whereas vision with both eyes yields no relief or perception of depth.

When one looks with a single eye at an image, or even better, at a photograph of a landscape or of objects positioned in perspective, the image seems indeed to have depth. Depth perception of objects is especially distinct for foreground objects, and it is favored by the sharpness of the outlines, and by the play of shadow and light. This is an easily explainable illusion; the perspective drawing evokes depth by association, which is so intimately related to its vanishing points and its play of light.

What remains to be explained is why the stereoscopic feeling vanishes as soon as one looks at the photograph with both eyes. The illusion does not endure, in the latter case, because in the photograph of a landscape, different objects give each eye a similar retinal image, contrary to what happens in the vision of real objects in which each object is painted on noncorresponding points of the retinas. Rather, the similarity and correspondence of retinal images are characteristics of binocular vision of flat surfaces. In binocular vision of a photograph, the present sensation of the flat surface thus negates the effects of the illusion of depth. In monocular vision, on the contrary, because this correction is absent, the field is left open for the illusion to play.

Perhaps another circumstance preventing the stereoscopic illusion from occurring could be found in the sensations of the convergence of the eyes that accompany binocular vision. Indeed, in binocular vision, the sensation of a flat surface is conditioned by the fact that when one’s eyes are scanning the photograph’s surface, the convergence angle remains the same, whatever point is fixated; any variation of convergence would produce diplopia. In monocular vision, diplopia is no longer to be feared; convergence of the open eye and the closed eye may thus be less precise, and it is likely that it varies slightly in a reflex mode, depending on whether one considers an object to belong to the first or last plane of the photograph. This convergence factor would thus have the effect of counteracting the illusion in binocular vision and encouraging it in monocular vision.

Monocular stereoscopic perception probably strikes all who have examined photographs with one eye (which is the case when looking through a magnifying glass), but I do not know whether this paradoxical phenomenon has been given the attention it deserves.

## Conclusion

Monocular stereopsis is a remarkable phenomenon that still remains to be explained unanimously. I have reviewed the history and theory of the phenomenon, provided biographical details of one of the research pioneers, Claparède, and provided a translation into English of his seminal 1904 paper.
